# Paper-based RNA detection and multiplexed analysis for Ebola virus diagnostics

**DOI:** 10.1038/s41598-017-00758-9

**Published:** 2017-05-02

**Authors:** Laura Magro, Béatrice Jacquelin, Camille Escadafal, Pierre Garneret, Aurélia Kwasiborski, Jean-Claude Manuguerra, Fabrice Monti, Anavaj Sakuntabhai, Jessica Vanhomwegen, Pierre Lafaye, Patrick Tabeling

**Affiliations:** 1 MMN laboratory CNRS UMR7083 Gulliver, ESPCI Paris, PSL Research University, Paris, France; 2 Institut Pasteur, HIV, Inflammation and Persistence Unit, Paris, France; 3 Institut Pasteur, Laboratory for Urgent Response to Biological Threats, Paris, France; 4 Institut Pasteur, Functional Genetics of Infectious Diseases Unit, CNRS URA3012, Paris, France; 5 Institut Pasteur, Antibody Engineering Platform, UtechS proteins, Paris, France

## Abstract

The most performing techniques enabling early diagnosis of infectious diseases rely on nucleic acid detection. Today, because of their high technicality and cost, nucleic acid amplification tests (NAAT) are of benefit only to a small fraction of developing countries population. By reducing costs, simplifying procedures and enabling multiplexing, paper microfluidics has the potential to considerably facilitate their accessibility. However, most of the studies performed in this area have not quit the lab. This letter brings NAAT on paper closer to the field, by using clinical samples and operating in a resource-limited setting. We first performed isothermal reverse transcription and Recombinase Polymerase Amplification (RT-RPA) of synthetic Ribonucleic Acid (RNA) of Ebola virus using paper microfluidics devices. We further applied this method in Guinea to detect the presence of Ebola virus in human sample RNA extracts, with minimal facilities (carry-on detection device and freeze-dried reagents on paper). RT-RPA results were available in few minutes and demonstrate a sensitivity of 90.0% compared to the gold-standard RT-PCR on a set of 43 patient samples. Furthermore, the realization of a nine-spot multilayered device achieving the parallel detection of three distinct RNA sequences opens a route toward the detection of multiple viral strains or pathogens.

## Introduction

The recent Ebola outbreak in Africa, which caused the death of more than 11,000 persons, has highlighted the importance of performing early disease diagnosis, to proceed in a timely manner to sanitary actions, such as isolation and treatment, minimizing the risk of infection^1^. The only approach that enables the detection of an infection in the very first hours after the symptoms onset is based on molecular biology, as antigen detection assays are less sensitive or specific compared to NAAT (Nucleic Acid Amplification Test). To be specific, after the infection, viral RNA levels increase logarithmically to reach between 10^4^ to 10^6^ copies/µL at day 3–5, a critical time for survival. With a detection threshold of 1 to 10 copies/µL^2, 3^, NAAT has the capacity of detecting the infection in the very first day, preventing risks of contamination. This capability does not exist with standard immunoassays.

During the Ebola outbreak, diagnostic tests were performed by the gold standard method for detection of RNA viruses which consists of RNA extraction followed with reverse transcription (RT) and PCR (real-time Polymerase Chain Reaction), to amplify the viral genome and identify the presence of the virus on suspect cases^4, 5^. This represents an important piece of information, but, with the existing technologies, RT-PCR has a long time-to-result and requires non-transportable, expensive equipment (such as GeneXpert^6^) and well-trained personal. Those are scarce in limited-resources countries such as Liberia, Guinea, and Sierra Leone. International help from Non-Governmental Organizations and the World Health Organization made the implementation of Ebola Treatment Centers and diagnostic laboratories possible in a number of affected areas but the delay in the global awareness concerning the outbreak scale resulted in a delayed and inadequate outbreak response. Although there are many proposals in the literature to develop NAATs in point-of-care (POC) devices^7–11^, the access of the population to NAAT diagnostics still raises challenging issues in terms of cost, consumable availability, transportability, sample preparation and simplicity of the operation mode.

From that perspective, paper microfluidics represents a promising technology. Paper microfluidics is a friendly-user, low cost technology, using paper as the solid matrix for managing the fluids in complex networks^12–15^. Until recent years, this technology has been applied to immunoassays. Nonetheless, with the development of isothermal amplification, it has recently served the identification of nucleic acid targets^16–19^ with techniques such as RT-RPA^9, 20^ which is particularly suitable for paper-based applications as its working temperature (between 37–42 °C) requires neither large thermal energy nor cycle control. Considering the chemical reactivity of paper^21, 22^, and the biochemical complexity of the amplification reagents, there was a serious risk that methods developed in the laboratory would fail when applied in the field. In the case of the Ebola virus (EBOV), due to the extreme contagion risk and constraining sanitary procedures, obtaining clinical samples was extremely difficult. By operating in a treatment center in Guinea, we were able to perform proof-of-concept tests on EBOV infected patient samples and thereby assess for the first time, the performances of a NAAT based on paper microfluidics for a viral contagious disease. Extending this work to multiplex detection is further discussed.

## Performing RT-RPA On Paper

**Figure 1:**
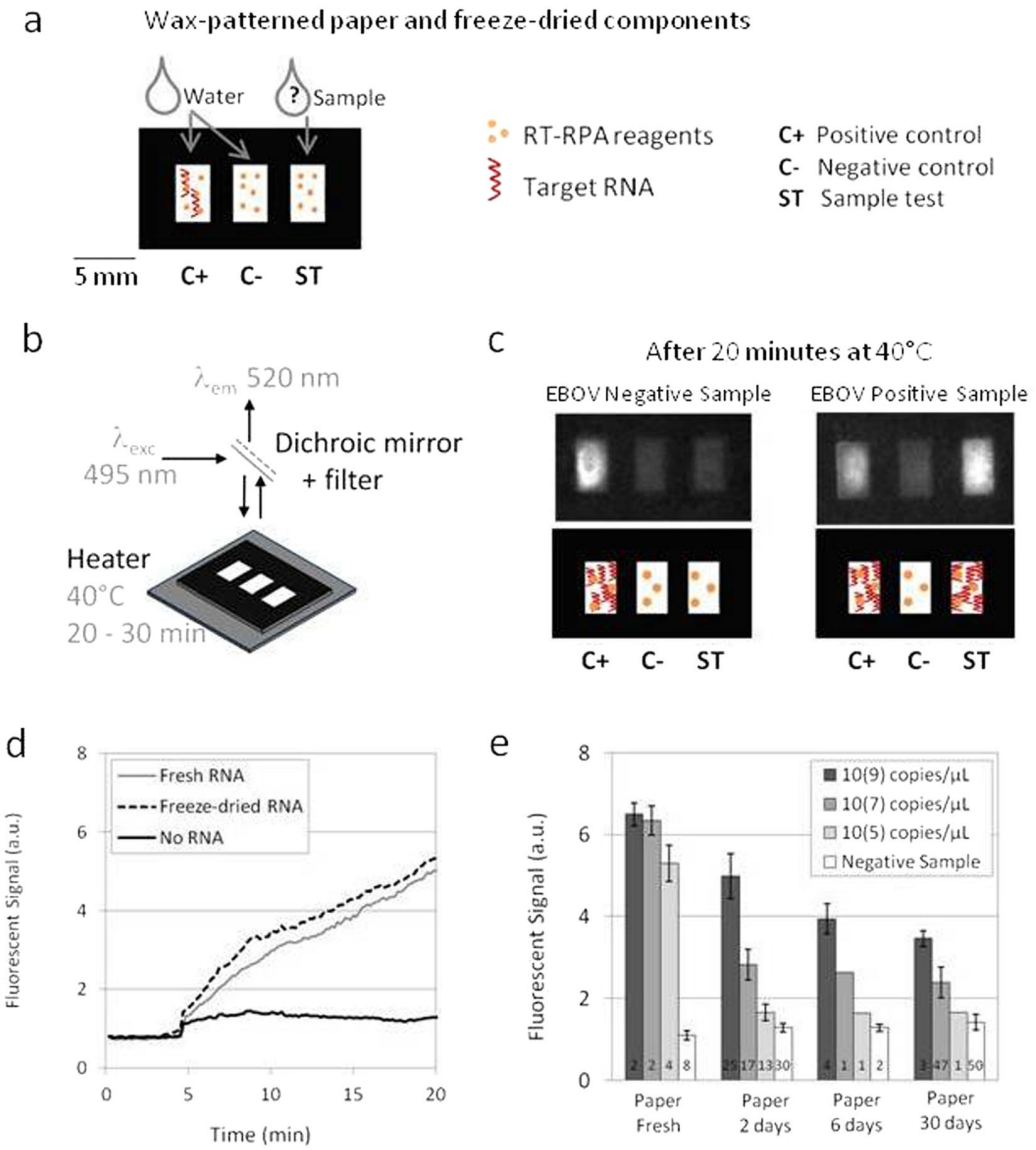
RT-RPA on paper. (**a**) Wax-patterned geometry on paper and location of freeze-dried RT-RPA reagents and RNA template, rehydrated either with water or the sample. (**b**) Scheme of the experimental set-up: the paper is put on a heating device and lighted with a 495 nm wavelength; the fluorescent recording at 520 nm is obtained after filtration of the emitted signal. (**c**) Pictures and scheme of typical results obtained with samples from a healthy donor and an EBOV infected patient, recorded from the experimental set-up presented in (b**)**. (**d**) Fluorescent signals recording over time for freeze-dried RT-RPA reactions performed on paper and rehydrated with 2.5 µL of distilled water (no RNA - black line), with 2.5 µL of 10^7^copies/µL RNA A (fresh RNA - grey line), with 2.5 µL of distilled water on a zone already containing the same quantities of freeze-dried RNA A (freeze-dried RNA - black dotted line). (**e**) Compared signal intensities for RT-RPA reactions performed with freshly prepared reagents on paper or with all reagents freeze-dried on paper and stored 2 days, 6 days or 30 days; with various concentrations of target RNA A (dark grey bars: 10^9^, medium grey bars: 10^7^, light grey bars: 10^5^, white bars: 0 copies/µL).

Ready-to-use micro-Paper Analytical Devices (µPADs) are prepared by freeze-drying RT-RPA mixture on individual paper areas. The experiment consists in rehydrating each spot with DNase/RNase-free distilled water with or without RNA template, heating the device at 40 °C and monitoring the fluorescent signal over time. Figure 1 shows paper design and experimental set-up to perform RT-RPA on paper, as well as main results obtained with synthetic RNA.

All paper devices are built with three rectangular areas corresponding to a sample test associated with two controls (positive and negative). As displayed in Fig. 1a, the three wax-patterned paper spots contain all the freeze-dried components of a RT-RPA reaction without the RNA matrix except for the positive control area. This spot additionally contains synthetic RNA corresponding to the region amplified by the primers that was freeze-dried together with the rest of the reagents. Controls are rehydrated with water and similarly to standard protocols, they ensure that there is no degradation of reagents and no source of contamination. Reaction in the third spot consists in a sample test when rehydrated with the RNA extracted from patient body fluids with an unknown EBOV status. After rehydration, the µPAD is placed in the experimental set-up which includes a heating device and the recording of fluorescence emission, as shown in Fig. 1b. Emissions of fluorescence on a µPAD, after 20 minutes of reaction, are displayed in Fig. 1c (movie available in Supplementary Video 1): only the positive control lights up with a sample from a healthy donor whereas both positive control and sample test produce fluorescent signals for a sample positive for EBOV.

Amplification plots consist in the recording of the fluorescent signal on each spot over time after rehydration, while heating at 40 °C. As shown in Fig. 1d, the RT-RPA test displays a clear rise in fluorescence with RNA either freeze-dried on the paper or freshly added for the reagent rehydration, as compared to the negative control. Other preliminary experiments about µPAD materials compatibility with RT-RPA reaction are presented in Supplementary Figures 1–3.

One important question concerns the storage of the ready-to-use µPADs, before use in the field. Time, temperature and humidity are factors to take into account as they might affect the subsequent RT-RPA reaction (Supplementary Figure 4). Results detailed in Fig. 1e present the comparison of amplifications performed from fresh reagents in tubes to those implemented with freeze-dried reagents on papers stored from 2 to 30 days, at room temperature, safe from light and humidity. The histogram displays the final fluorescent intensity (after 20 minutes of reaction) measured from tubes and paper areas, after amplification of different concentrations of synthetic RNA templates. Negative controls on paper are demonstrating a higher fluorescence value than in tubes due to the white color background of paper. Still, detection of amplifications is possible for high concentrations of synthetic RNA (10^9^ and 10^7^ copies/µL). The limit of detection on paper appears to be 10^7^ copies/µL which is quite higher than in tubes (lower than 10^5^ copies/µL). Although time of µPAD storage affects the signal intensity, the sensitivity remains the same even with freeze-dried reagents stored for one month. After three months, 20% of the paper devices remain operational (not shown). Note that the concentrations we discuss here are high compared to other molecular detection methods and far above the required limit-of-detection (around 10^1^ copies/µL) in the case of EBOV diagnostics: there are reached only in fatal cases^2, 23^.

## On-Field Experiments

**Figure 2:**
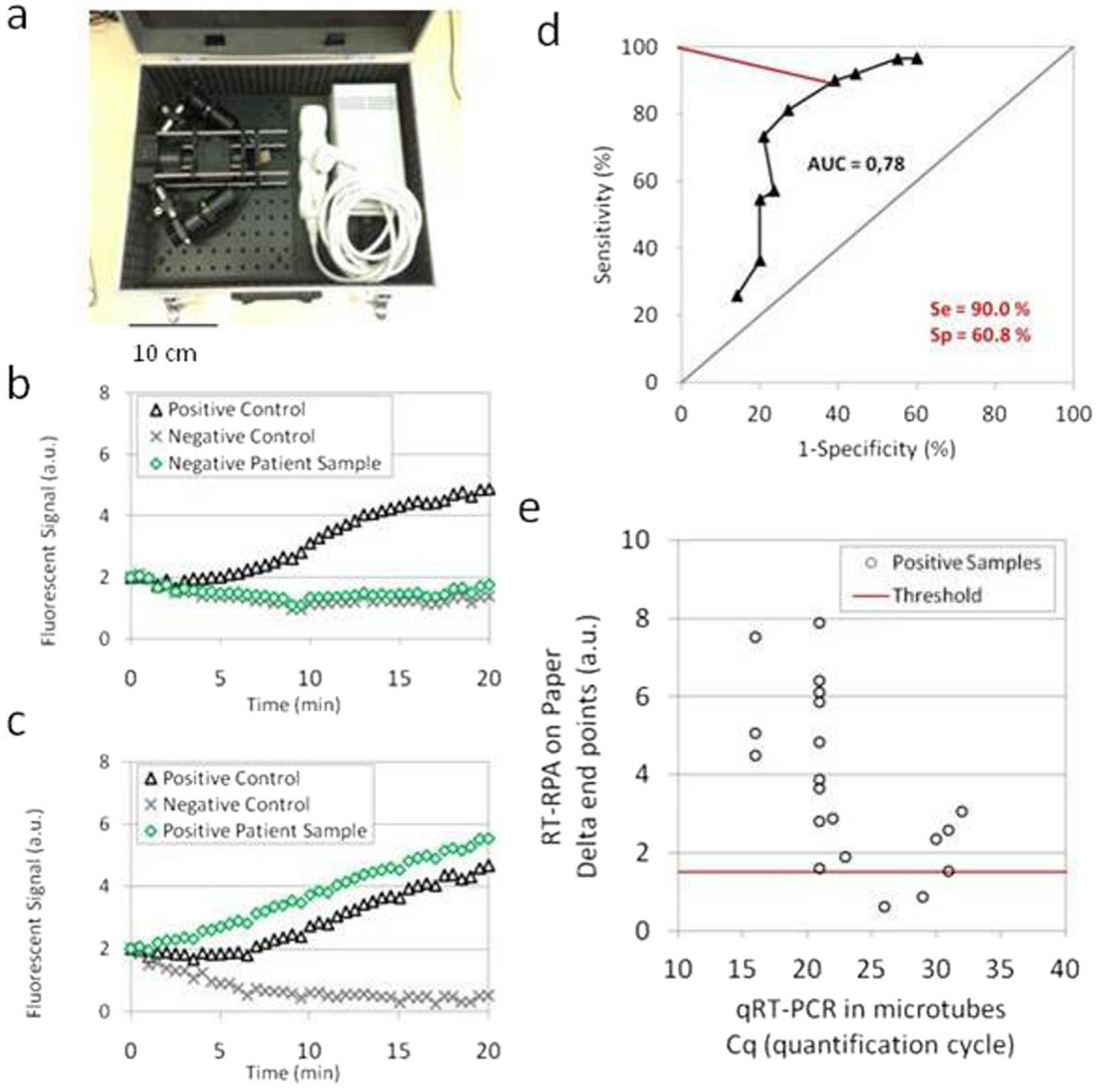
Experiments in Guinea. (**a**) Picture of the transportable equipment: heating device and optical system of detection fixed in a suitcase. (**b**) Amplification curve for a negative patient sample (green diamonds) and its related positive (black triangles) and negative (grey crosses) controls measured with the instrumentation presented in (a). (**c**) Amplification curve for a positive patient sample (green diamonds) and its related positive (black triangles) and negative (grey crosses) controls measured with the instrumentation presented in (a). (**d**) ROC curve related to sample tests (n = 43) analysis only when positive/negative controls on the same paper well give the expected results (black triangles). The Area Under Curve (AUC) parameter is calculated to 0.78 and a selected threshold (in red) gives a sensitivity and a specificity respectively to 90.0% and 60.8%. (**e**) Compared results between RT-RPA on paper (end value difference between the sample test and the negative control) and qRT-PCR in microtubes (quantification cycle) for positive Guinean samples (black rings) (n = 20). The red line represents the threshold value determined in (d).

We built a transportable set-up (Fig. 2a) to perform tests in a minimal settings environment. All elements are anchored in a carry-on suitcase. The sensitive camera and powerful UV lamp are replaced with simple and small USB linear camera and two LEDs. Supplementary Figures 5–9 gather some additional results to compare the two detection devices.

In August 2015, this equipment was used in Macenta (Guinea) where the French and Guinean Red Crosses had settled an Ebola Treatment Center and a small laboratory operated by the Institut Pasteur during the 2014 outbreak. In this laboratory, RNA had been extracted from the plasma of EBOV infected patients and stored at −20 °C. We tested these samples on our paper devices. Ready-to-use papers, with the same geometry as presented in Fig. 1a, were prepared in France and stored in small black packages with desiccant, in order to protect them from light and humidity. Positive controls contained a freeze-dried 10^7^ copies/µL synthetic RNA template. They were then sent to Guinea, during the rainy season (mean temperature and humidity are respectively recorded at 26 °C and 87–91%), without further precautions and stored in the air-conditioned laboratory, during one month prior to running experiments. Figure 2b and c display examples of amplification curves obtained from the sample of, respectively, a healthy donor and an EBOV positive patient. Positive controls display clear fluorescent signals rise when temperature is increased to 40 °C whereas the negative ones do not change significantly. It is remarkable that RT-RPA analysis on paper only takes 20 minutes against 110 minutes for the RealStar® Ebolavirus RT-PCR kit from Altona, considered the gold standard method for EBOV RNA detection. This time saving can be of crucial importance to quickly proceed to patient triage and treatment.

The positive or negative status of a sample is provided by the result of the RT-PCR carried out on the day of the patient inclusion. However it should be kept in mind that even this method does not have optimal performances (a question raised in ref. 24). Moreover, our RT-RPA test on paper was performed up to 8 months afterwards on RNA extracts stored at −20 °C instead of −80 °C which is known to be a better temperature for RNA storage especially for low RNA quantities^25^. These conditions can have affected our experiment results as it was not possible to check again for the viral RNA left in the samples with a standard RT-PCR at the time of µPAD test. Note that in Guinea, it has not been logistically possible to realize blind experiments. Our results from a set of 50 samples from Guinea – and 18 additional healthy donors from France to complete the pool of negative templates – must therefore be considered as a first-stage proof-of-concept and not a state-of-the-art validation.

For each amplification curve, we calculate the end point (after 20 minutes at 40 °C) intensity difference between the sample test and the negative background. The distribution of this parameter for all experiments is available in Supplementary Figure 10. The same analysis is performed between positive and negative controls, to insure correct functioning of the paper devices. The question is whether it is possible, from these measurements, to define thresholds above which the sample can be declared positive. The ROC (Receiver Operating Characteristic) curve that simultaneously displays sensitivity and specificity of the device, for each value of a threshold (Fig. 2d) can provide answers to this question. Sensitivity is defined as the ratio of infected patients that are well-diagnosed by our device whereas specificity represents the rate of non-infected patients with a negative result on paper tests. When only sample tests with validated internal controls are taken into account, the Area Under Curve parameter reaches 0.78. With an arbitrary threshold, our diagnostic tool displays a high sensitivity (90%) without unduly damaging the specificity (60.8%). Still with this threshold, amplification results measured on paper can be compared with the quantification cycle values obtained by RT-PCR in microtubes (Fig. 2e). Highly concentrated samples (low quantification cycle) can be easily detected by RT-RPA on paper whereas less concentrated samples (high quantification cycle) present a RT-RPA parameter closer to the threshold. However, there are only two false negatives among 20 tests, and we succeeded in detecting all the 4 clinical samples necessitating more than 30 cycles of RT-PCR for its detection. For these samples, the fluorescent signal was efficiently detected only after a time longer than 15 minutes. Thus, longer reaction time might be necessary for low viral load detection on paper as compared to RT-RPA in tubes. According to a comparative study^26^, quantification cycles above 30 with Altona kits are obtained with RNA concentrations lower than 10^2^ copies/µL enabling an early diagnostics within the very first days after symptoms onset^2, 23^. RT-RPA-on-paper reaches satisfying diagnostic performances with viral RNAs, while in the laboratory, for unknown reasons, sensitivity on synthetic RNAs was found more modest. This observation underlines the importance of performing tests on clinical samples.

## Multiplexed Paper Devices

**Figure 3:**
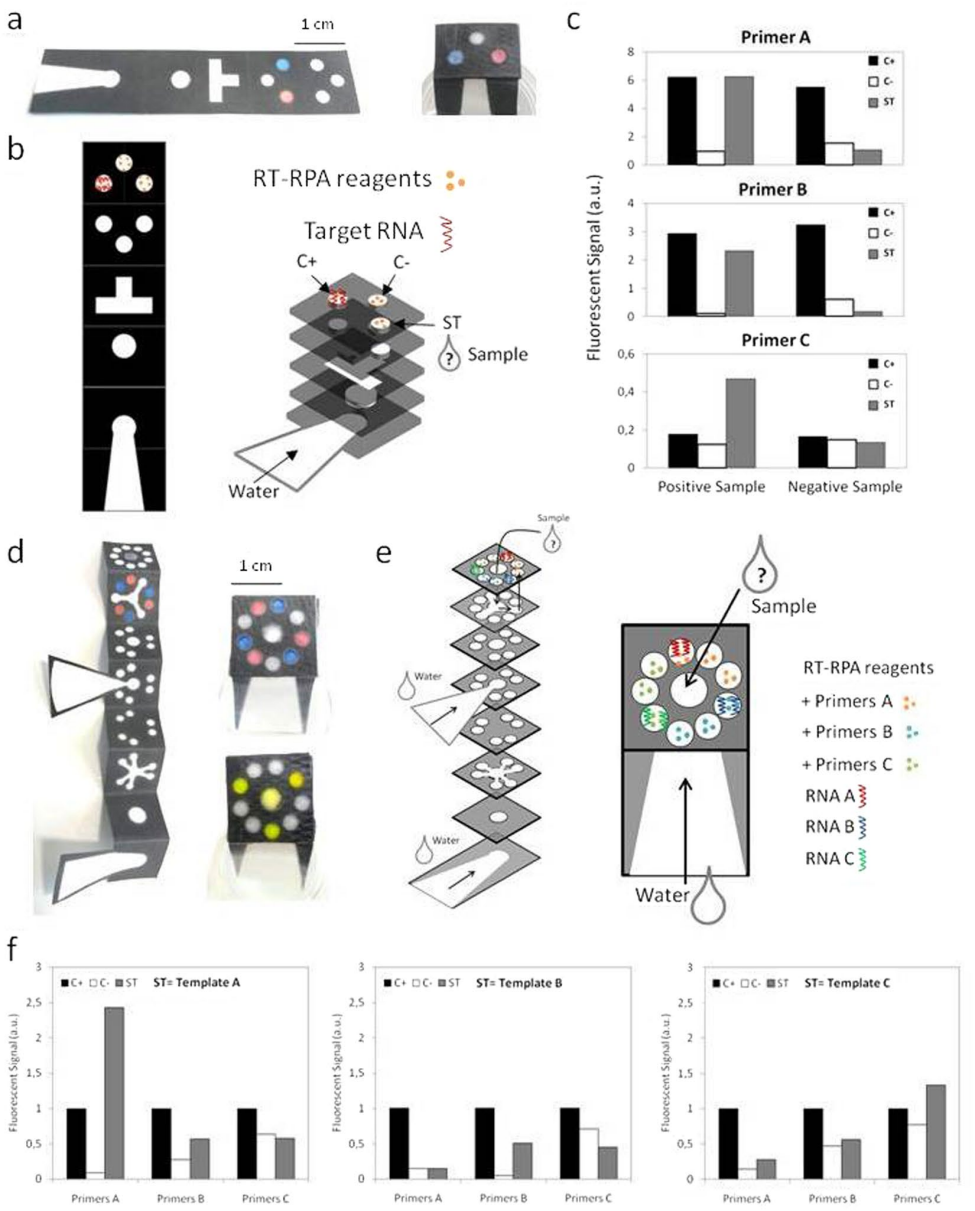
Multiplexed paper devices: performing several simultaneous reactions thanks to liquid flows. (**a**) Picture of an unfolded wax geometry (multilayered geometry), with dry amounts of dyes in the second last layer. Picture of the same device after folding and dipping it in water: the dyes are conveyed to the top without mixing together. (**b**) Schematics of the unfolded multilayered wax geometry and location of freeze-dried RT-RPA reagents and targeted RNA template. Exploded view of the folded device: the paper tape should be dipped into water and sample pipetted in the sample test outlet. (**c**) Results of amplification (final intensity value) for each set of primers (A, B or C) with or without the related RNA template in the sample test outlet (grey bars), with associated controls (positive: black bars; negative: white bars). (**d**) Picture of an unfolded longer wax geometry (multiplexed device), with dry amounts of dyes in the second last layer. Picture of the same device after folding and dipping into water: the dyes are conveyed to the top without mixing together. Picture of the same device, without dry components stored in it, fluorescein is pipetted in the middle spot simultaneously to the strip dipped in water. The yellow dye is distributed towards three sample tests outlets. (**e**) Schematics of the multiplexed paper device: exploded view and top view, and location of freeze-dried biological components (RT-RPA reagents with three different primers and associated RNA templates). (**f**) Normalized compared results of specific amplification of each template in the 9-outlet device (positive controls: black bars, negative controls: white bars; sample tests: grey bars).

Multiplexed diagnostics is a strong request from international public health organizations and can be used in several ways: to simultaneously detect several pathogens that are responsible for similar symptoms, to improve the sensitivity towards one disease by targeting several biomarkers or RNA strains, to get more information about a pathogen like identifying mutants. For this purpose, paper microfluidics has the full potential to meet the goal^27, 28^. In the context of NAAT, only one publication refers to parallelized analysis but still with several manual pipetting operations^29^. We made a five-layer paper device (Fig. 3a and b). This design allows analyzing one sample along with visualizing positive and negative controls simultaneously, using a minimal procedure: dipping the device into DNase/RNase-free water and pipeting the sample on paper. The design we used simplifies the preparation and reduces distances that fluids have to travel to reach their targets. In fact, the design of Fig. 3a and b benefits from a valve-like function induced by the folding: when folded, we establish a fluidic connection between two adjacent layers, in a way similar to opening a valve in a microfluidic device. In our case, the reagents are first freeze-dried on isolated circular areas on the unfolded patterned paper sheet. Then, the sheet is folded to form a ready-to-use five-layer paper device.

Experiments with dyes allowed checking the fluidics of devices. As shown in Fig. 3a, water rehydrates and conveys the dyes towards their targets, i.e. the circular zones accessible to optical observation. The neat and unblended aspect of the colors confirms that no cross contamination occurred. This important result is consistent with estimates based on the Peclet number^30^. Specifically, the distance between two outlets is approximately 10 mm and the liquid speed is approximately 0.1 mm/s. For mobile species, the diffusion coefficient can be estimated to 10^−6^ cm²/s. This leads to Peclet numbers of approximately 10^4^, from which we conclude that diffusion can be neglected and thereby, owing to the simplicity of the geometry, no mixing occurs during the imbibition of the device by water (hydration step). During the reaction step, the device is heated at 40 °C and one must wait 20 minutes for the amplification to be completed. Still, cross contamination is negligible during this step because, owing to diffusion-based estimates, it would take 45 hours for the reagents to reach the adjacent well, which is comfortably longer than the test duration. Note that when the device is fully imbibed, fluids are still moving, owing to the action of evaporation. This process, being associated to moderately high Peclet numbers, works against cross-contamination, and reinforces the precedent argument. Amplification results for three sets of primers are displayed in Fig. 3c and Supplementary Video 2. Although the difference is thin in primers C case, positive and negative controls well display different behavior that helps diagnosing the sample status.

We applied the same approach to the fabrication of a 9-outlet multilayered paper device shown in Fig. 3d, illustrating the concept of multiplexing the diagnostics of three diseases, again with positive and negative controls at display. Here we used three sets of primers freeze-dried on the paper, that represent, coupled with corresponding Ebola synthetic RNA target, three distinct biological samples. This experiment, beyond illustrating the feasibility of multiplexing the detection of diseases on paper devices, may be taken as a method for optimizing the choice of primers. The device preparation is illustrated in Fig. 3e. An exploded view of the folded device enables to see the stack of paper layers. When paper strips are dipped into DNase/RNase-free distilled water, the liquid flow from the bottom layer rehydrates reagents on the nine outlets. The fresh sample is added in the sample test outlet. Figure 3f displays the results of amplification for each template in this 9-outlet multilayered paper geometry. A movie is also available in Supplementary Video 3. For better reading and primer comparison, intensity values are normalized to fix each positive control to 1. Negative controls define the background level. In the sample test areas, although there is a bit of systematic and non-specific signal with primers B, amplification occurs only in presence of the template RNA.

To conclude, the present work brings NAATs on paper close to on-field applications. We show that reagents can be freeze-dried, stored, transported close to the point-of-care. Despite its biochemical complexity – RNA extracts and RT-RPA reagents are brought together in the same cellulose well – the system is performing satisfyingly as the sensitivity of RT-RPA-on-paper technique was of 90.0% compared to the gold standard RT-PCR for results obtained on 43 clinical samples. Moreover, we figured out a design that simplifies the device preparation and minimizes the number of manual operations to perform three simultaneous analyses. Our approach can be extended to other viral diseases such as HIV and dengue fever, or other microorganisms, such as malaria^31^. Nonetheless, sample preparation, i.e. RNA extraction on filtered blood, remains a challenge. Recent work indicates that solutions with minimized equipment exist, such as the paper machine proposed by Diagnostics for All^18^ and other approaches are currently discussed^10, 14, 32–38^. Next steps in the development of this POC test would be either add an RNA extraction kit system in the same suitcase, as it has been shown before^39^ or to integrate a paper filter that allows RNA extraction in the same device^19, 29, 34, 40^. Such device has been developed for pathogens with DNA genome which is known to be less sensitive to degradation than RNA^18^. Plasma separation membrane or filter microchip could also be considered^41–43^. In that case, RT-RPA would be done without RNA extraction but would need a preheating step to 92 °C to release the RNA from the viral particles as shown in a standard RT-PCR method^35^. This high temperature implies a separate system to not alter the RT-RPA reagents. Moreover, colorimetric or minimally-equipped solutions are currently described in the literature^20, 29, 44^ and should help release the equipment-dependence. Whatever the solution may be, it is important to show that in real conditions, paper does not introduce biases, detrimental effects, nor performance degradation and its cost remains potentially low, although paper represents only a part of the device. By showing that these artifacts do not exist, along with figuring out a dedicated multiplex design, we single out a pathway that may bring, nucleic acid based diagnostic on the field thanks to its simplicity and affordability.

## Methods

### Ethics statement

According to the Helsinki Declaration (http://www.wma.net/en/30publications/10policies/b3/index.html), no informed consent from the patients was required due to the exceptional circumstances of the Ebola virus outbreak during this period. This study has been evaluated and approved by the ethics committee of Guinea (ref: 99/CNERS/16), the Ebola research committee and the Institutional Review Board (ref. 2015–012) at Institut Pasteur. The subject information was anonymized prior to analysis. All methods were performed in accordance with the approved guidelines.

### Sample collection and processing

50 samples were collected from suspected Ebola cases hospitalized at an Ebola treatment centre in Macenta, Guinea. EBOV detection was tested by quantitative RT–PCR (RealStar® Ebolavirus RT-PCR kit from Altona) using a Taqman assay with 5-FAM and 3-TAMRA probes on a portable Smart-Cycler TD. Each sample was run three times on three separate assays and compared with our experiments. 34 of these samples with a positive RT-PCR result presented a Quantification Cycle representative of the whole Ebola positive population. 18 additional negative samples obtained from the plasma of healthy donors were performed in a second time in the laboratory to reach a pool of 68 samples (34 positives and 34 negatives). RNA extraction was done with the same kit (Qiagen, Hilden, Germany) and internal controls (Altona, Hamburg, Germany) than those used in Macenta.

### RT-RPA reagents

RT-RPA assays specific for Ebola virus were developed. Three conserved regions of the Zaïre virus genome were selected based on the alignment of 145 published sequences^45^. They spanned nucleotides 8560–8929, 16897–17284, 1657–2089 for region A, B and C respectively. These regions were cloned on pRSET plasmid under T7 promoter (GeneArt, Thermofisher, Waltham, MA, USA). *In vitro* transcription allowed to obtain the corresponding synthetic RNA (Thermofisher). Primers and probes, labeled A and B, specific to each template A or B, were designed following the RT-RPA kit manufacturer recommendations. Sequences for primers and probe labeled C, come from the literature^45^. Here are summarized the sequences of the forward and reverse primers, and probe for each region as well as the size of the amplification products: [A/5′-CTA CTG TAT TTC ATA AGA AGA GAG TTG AAC C-3′/5′-AAT TGT TGT TCT ACT GAT CCA CAA GTC TTA C-3′/5′-ATA TGT CCG ACC TTG AAA AAA GGA TTT TTG [FAM-dT][THF][BHQ1-dT] GAC AGT AGT TTT TGC [3′-phosphate]/160 bp]; [B/5′-CTA CTG AGT CCA GTA TAG AGT CAG AAA TAG TA-3′/5′-CTG AGT TGT TAA GAA TAA TCT CAA TTT GGT-3′/5′-AAT GAC TAC TCC TAG GAT GCT TCT ACC TGT [FAM-dT][THF][BHQ1-dT] GTC AAA ATT CCA TAA [3′-phosphate]/127 bp]; [C/5′-GAC GAC AAT CCT GGC CAT CAA GAT GAT GAT CC -3′/5′-CGT CCT CGT CTA GAT CGA ATA GGA CCA AGT C -3′/5′-GAT GAT GGA AGC TAC GGC GAA TAC CAG AG [FAM- dT] T [THF] C [BHQ1-dT] CGG AAA ACG GCA TG [3′-phosphate]/168 bp]. When not mentioned, template A, primers and probe A were used. RT-RPA was performed using a TwistAmp RT-Exo kit (TwistDX, Cambridge, United Kingdom), as previously described^31^. The result of amplification can be followed by fluorescence thanks to the exonuclease activity that unquenched the probe in case of hybridization. All reagents except the sample were mixed, distributed on paper at −20 °C and freeze-dried for 1.5 hours. Ebola viral RNA extracted from patients plasma were tested with this method.

### µPADs fabrication

Micro-Paper Analytical Devices (µPADs) consist in Whatman grade 1 chromatography paper printed with wax^46^ and baked 1 min at 150 °C on a Ika RCT basic heating plate. Patterns were designed with CleWin5 software and printed with a classic wax printer Xerox 8580 ColorQube. Multilayered devices were fabricated by designs lining up and paper folding. A good contact was obtained by sealing each layer with Tesa doubled-stick tape put around the flow patterns. RT2RR Sigma plastic foils enable to close and isolate the µPAD. 5 mm holes were punched in the plastic foil to give access to inlets and outlets. Several layers of pierced plastic foils form a liquid reservoir that can be closed with an additional non-punched plastic foil, during the experiments, to prevent from undesired evaporation.

### µPADs geometry

Three designs were considered to demonstrate the feasibility of RT-RPA on paper: individual paper spots and two multilayered geometry. A single deposit area was obtained by a rectangular shape: length 5 mm and width 3 mm. Three rectangles spaced by 2 mm fit the linear camera field. A first multilayered geometry consists in a paper strip that can dip into water. Water flows are distributed towards three areas: sample testing, positive and negative controls. A second multilayered geometry can perform three simultaneous RNA tests and their respective positive and negative controls. Water is pumped through a paper strip at the bottom layer. The following layer enables to distribute the flow towards six controls outlets and to the sample inlet. The sample can be brought from the top of the device, in the middle inlet. It is then distributed towards three outlets. Experiments with dyes enable to visualize the flow: no mixing between all outlets and the sample trajectory. Fluorescein dye is purchased from Reactifs RAL and used at 10^−3^ g/mL concentration. Brilliant Blue G and Allura Red AC used at 10^−3^ g/mL are purchased from Sigma Aldrich.

### Experimental procedure

RT-RPA freshly prepared mixture was put on the desired paper area: on individual rectangular spots for the first geometry or in the outlets of multilayered devices. After freeze-drying, µPADs were folded (only for multilayered geometries) and sealed with plastic foils. They were stored at room temperature, safe from humidity and light, during one day to several weeks. Then the experiment consisted in bringing sample and water to the single deposit area, and closing the system with an additional foil. For multilayered devices, the sample was brought in the specific deposit area, closed with plastic foil, and the paper strip was dipped into DNase/RNase-free distilled water. The µPAD is then heated to 40 °C thanks to a heating device during 20 to 30 minutes.

### Signal recording and analysis

Quantitative fluorescent detection was performed for each experiment with Leica EL 6000, an external light source for fluorescent excitation, and with Leica Z16 APO macroscope, magnification ×0.57, with L5 Leica filter cube. This filter contains an excitation filter at 480 nm ± 20 nm, a dichroic mirror at 505 nm and a suppression filter at 527 nm ± 15 nm. These optical conditions suit well the FAM probe spectral properties (absorption wavelength: 495 nm, emission wavelength: 520 nm). Images were recorded from Hamamatsu EM-CCD Camera C900-13, one image every 10 seconds during 20 minutes, with an exposure time of 75 ms. Synchronization between shutter aperture and camera acquisition prevents from sample bleaching, and was realized thanks to EG R&D Vision delays generator. ImageJ freeware was used to normalize data by subtracting the first image to each image sequence. Signal over time is given by an average signal measurement on the test area, for each image.

### Carry-on equipment

On-field experiments were realized thanks to carry-on detection equipment. It consists in two blue LEDs (Thorlabs – M490L3–490 nm) combined with a first lense (Thorlabs ACL25416U-A), two filters (Thorlabs FES0500 – Thorlabs M497-16) and a second lense (Thorlabs LA1422-A) to light the paper. The signal emitted by the biological reaction on paper is collected by a linear camera (Thorlabs LC-100) through a first lense (Thorlabs ACL3026-A), a FITC dichroic filter (Thorlabs MD499), a filter (Thorlabs FELH0500) and a second lense (Thorlabs ACL25416U-A). The paper device is positioned on a chip holder heated by a PTC heater (DBK HP05), controlled by a platine thermic element (RS Components PT 1000ohms) and a temperature controller (Carel IR33). A home-made Microsoft Office 2007 macro program enables to extract data from the linear camera recording software (Splicco), to detect the position of the maximum fluorescence intensity for each paper rectangular area and to monitor the mean intensity around each maximum over time.

## Electronic supplementary material

Supplementary Information
