# Supplementary material for: Paper-based RNA detection and multiplexed analysis for Ebola virus diagnostics
Source: Sci Rep. 2017 May 2;7:1347. doi: 10.1038/s41598-017-00758-9 (PMC5431003; doi:10.1038/s41598-017-00758-9)
Supplement: Supplementary file 1 — Supplementary Information [file 41598_2017_758_MOESM1_ESM.pdf]

Paper-based RNA detection and multiplexed analysis for Ebola virus diagnostics – Supplementary Materials

Laura Magro[1], Béatrice Jacquelin[2], Camille Escadafal[3], Pierre Garneret[1], Aurélia Kwasiborski[3], Jean-Claude Manuguerra[3], Fabrice Monti[1], Anavaj Sakuntabhai[4], Jessica Vanhomwegen[3], Pierre Lafaye[5], Patrick Tabeling[1]*

[1] MMN laboratory CNRS UMR7083 Gulliver, ESPCI Paris, PSL Research University, Paris, FRANCE.
[2] Institut Pasteur, HIV, Inflammation and Persistence Unit, Paris, FRANCE.
[3] Institut Pasteur, Laboratory for Urgent Response to Biological Threats, Paris, FRANCE.
[4] Institut Pasteur, Functional Genetics of Infectious Diseases Unit, Paris, FRANCE / CNRS URA3012, Paris, FRANCE.
[5] Institut Pasteur, Antibody Engineering Platform, UtechS proteins, Paris, FRANCE.
*Correspondence should be addressed to patrick.tabeling@espci.fr

Supplementary Video 1 online: RT-RPA amplification in paper microzones.
https://www.dropbox.com/s/fbupjylchd6twge/S1%20-%20SUPPLEMENTARY%20MATERIALS%201.avi?dl=0

Supplementary Video 2 online: RT-RPA amplification in paper multilayered device with 3 outlets.
https://www.dropbox.com/s/sdu0pmum7ggyysu/S5%20-%20SUPPLEMENTARY%20MATERIALS%205.avi?dl=0

Supplementary Video 3 online: RT-RPA amplification in paper multilayered device with 9 outlets.
https://www.dropbox.com/s/sy7w4cg89f7nxln/S6%20-%20SUPPLEMENTARY%20MATERIALS%206%20no%20sample.avi?dl=0

Fluorescent recordings of RT-RPA on three paper designs. All the movies were recorded with the experimental set-up presented in Fig. 1b.

In Supplementary Video 1 online, three individual rectangular wax-patterned paper spots contain freeze-dried RT-RPA reagents, and the additional RNA template for the positive control. The paper device is enclosed between several layers of plastic foil: one on the back side and three pierced layers (visible with circular shapes) on the top side to form a reservoir and to let a free access to the paper spots. After manual rehydration of each spot (water for controls and target RNA for the sample test), a final plastic foil is used to enclosed the device and prevent from evaporation. As the volume of each rehydration droplet is larger than the accessible volume in the porous media, a part of the liquid is confined between the paper surface and the closing plastic foil. When amplification occurs, there is a larger contribution of this part of the droplet to the fluorescent signal. This explains the non homogenous distribution of the fluorescent signal over each paper spot.

The Supplementary Video 2 online displays the top layer of the three-outlet multiplexed paper device. A pierced plastic foil is visible on the top layer and ensures good sealing between layers in the folded device. However, in this case, it is not necessary to isolate the reaction areas with a final plastic foil. As previously, all the spots contain RT-RPA reagents, and the additional RNA template for the positive control. The template RNA is pipetted in the Sample Test area whereas controls are rehydrated by liquid flows from the paper strip and the internal layers of the µPAD. Signal of amplification in the positive control and in the sample test in presence of the target RNA does not homogenously appear. Still a recording of the mean fluorescence intensity value over the whole surface of the paper spot enables to easily distinguished negative from positive results.

An experiment with the nine-outlet multiplexed paper device is presented in Supplementary Video 3 online. With this geometry, no plastic foil was used. The layers were just assembled with double side tape. Some post-process of the video enables to better localize and identify each reaction spot: positive controls (C+), negative controls (C-), sample tests (ST) and the nature of the primers (A, B or C) in the freeze-dried reagents. All the spots are initially rehydrated by the liquid flow from the paper strips and the inner patterns. In this example, no sample was added; it simply demonstrates a negative case were only positive controls light up. The Fig. 3f illustrates other cases where either a RNA template A, B or C is pipetted in the central paper inlet. According to the sequence of these primers A, B and C, each sample test area can detect the same pathogen (to increase sensitivity, to screen several regions or to give more information about mutations and variants) or various infectious targets (Ebola virus, Zika, HIV …).

RT-RPA biocompatibility with µPAD components. A preliminary study has been done to check the biocompatibility of main µPAD materials towards RT-RPA reaction. Inhibitory effects of nucleic acids amplification are still not well understood but they can occur at different levels such as: the availability and integrity of nucleic acids or the enzyme activity (Williams, D. The Williams dictionary of biomaterials. *Liverpool University Press* (1999) - Williams, D. On the mechanisms of biocompatibility. *Biomaterials* **29**, 2941-2953 (2008)). Especially documented in the forensic field, exhaustive list of inhibitors and facilitators of amplification are available in the literature (Wilson, I. Inhibition and facilitation of nucleic acid amplification. *Appl. Environ. Microbiol.* **23**, 3741-3751 (1997)). However, a given material can have opposite influence in different examples.

Previously to the RT-RPA amplifications on paper, the biocompatibility of each µPAD component is individually checked. Experiments consist in standard RT-RPA reaction with positive or negative sample performed in microtubes (Supp. Fig. 1).

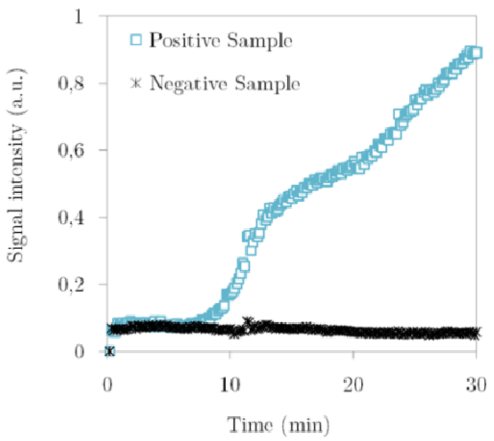

Supplementary Figure 1 *- RT-RPA in microtubes. Amplification curves with positive (blue squares) or negative (black crosses) samples.*

The same experiment is then performed with an additional piece of material (approximately 1mm^3) introduced in the microtube during the whole reaction (Supp. Fig. 2). The three materials tested are main components for µPAD fabrication: Whatman paper, wax used by the inkjet printer, plastic foils. The amplification curve from positive samples and the negative background measured with negative samples can be compared with regular RT-RPA reactions without additional material. None of these components are responsible for neither unspecific increase of the background signal nor high inhibition of amplification. Wax seems to delay a bit the amplification start and reduces the final end point value. The unknown composition of this commercial wax prevents from a good control of the biochemical environment. However the inhibitory effect remains very slight.

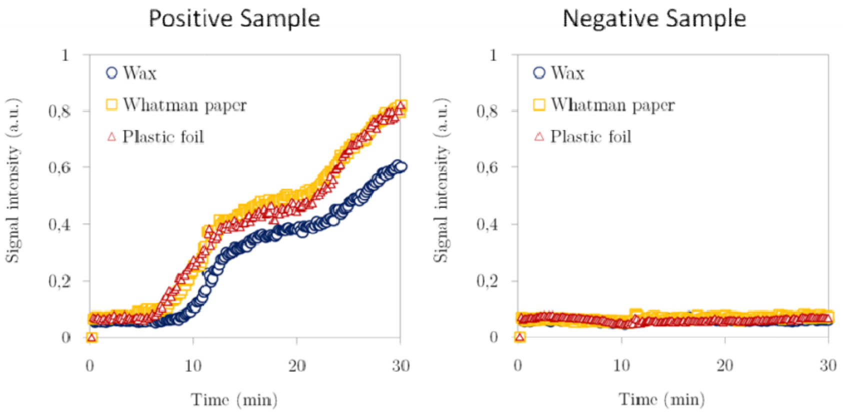

Supplementary Figure 2 *- RT-RPA in microtubes with an additional materials : wax (blue circles), Whatman paper (yellow squares), plastic foils (red triangles). Amplification curves with positive (**left**) or negative (**right**) samples.*

Supp. Fig. 3 presents the RT-RPA amplification curves obtained from fresh reagents and performed in tubes or on paper, with in both cases water as negative samples and synthetic RNA template (concentration at 10^7 copies/µL) as positive samples. Similar fluorescent intensities are measured on paper and in tube with positive samples. However, a higher negative sample displays a higher background level on paper than in tube.

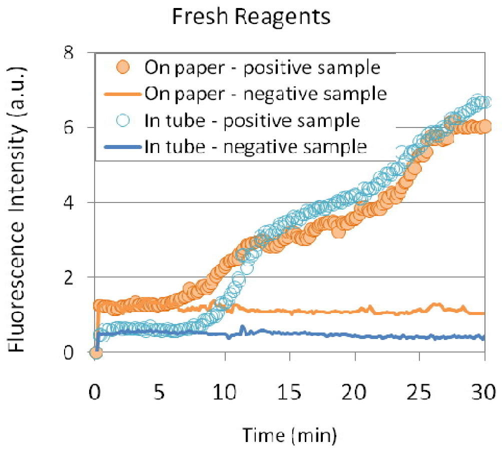

Supplementary Figure 3 *–RT-RPA assay with fresh reagents in tube (blue) and on paper (orange) with 2.5 µL of water (crosses) and with 2.5µL of template RNA concentrated at 10^7 copies/µL (circles).*

After freeze-drying reagents on paper, various storage conditions were tested with parameters such as temperature, pressure and humidity (Supp. Fig. 4). Amplification curves display fluorescent intensities of RT-RPA reaction on paper without RNA (negative control), with freeze-dried RNA (positive control) and with fresh RNA (positive sample test). Paper devices were enclosed in a sealed bag. At ambient temperature and in a dry environment, vacuum conditions cause a slight reduction of the intensity of amplification curves. Under vacuum and in a dry environment, a temperature rise to 30°C largely inhibits the amplification of fresh RNA when compared with ambient temperature (21°C). When combining a high temperature (30°C), vacuum conditions and a wet environment, no amplification occurs.

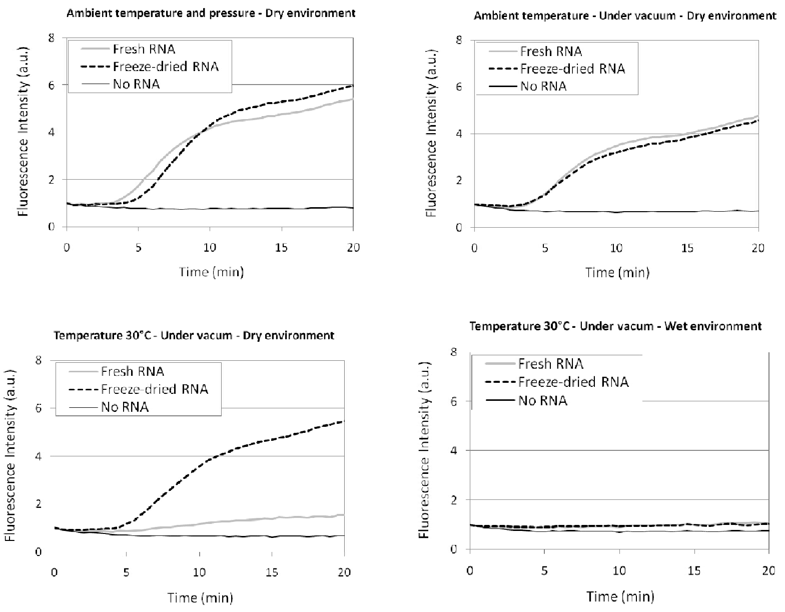

Supplementary Figure 4 *–RT-RPA assay on paper from freeze-dried reagents, with no RNA (black line), fresh (grey line) or freeze-dried (black dotted line) RNA template (2.5µL - concentration: 10^7 copies/µL). Paper and reagents were stored two days in various conditions: at ambient temperature or at 30°C ; in a small bag at ambient pressure or under vacuum, bag kept in a dry environment or dipped into water.*

Experimental set-up comparison. RT-RPA amplification signal on paper has been measured thanks to two detection devices. Initial laboratory developments and multiplexed analysis were done with very sensitive equipment described in the Methods section and presented in Supp. Fig. 5. The camera field of 2cm x 2cm enables to take picture of the three paper geometries that were used (Supp. Fig. 5c).

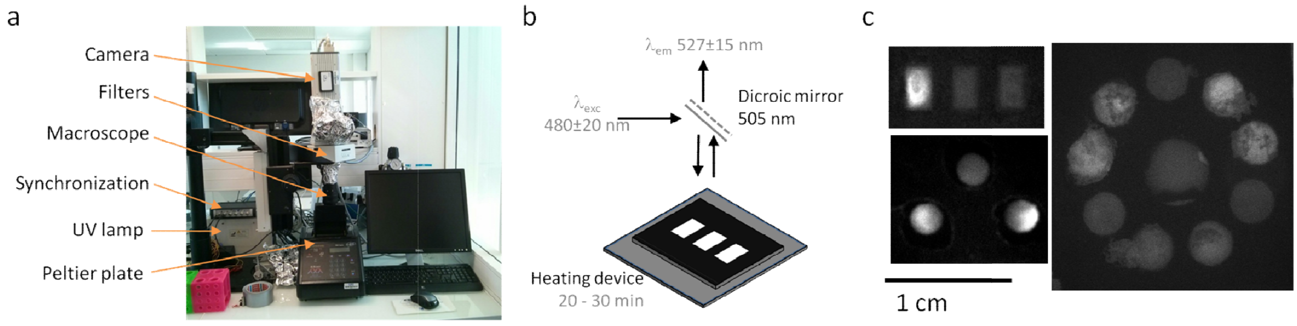

Supplementary Figure 5 *–Laboratory experimental set-up. (**a**) Picture of the instrumentation to perform the two functions: heating and fluorescence detection. (**b**) Scheme of the fluorescence detection with technical specifications: excitation (λ_{exc}), emission (λ_{em}) and cut-off (dicroic mirror) wavelengths. (**c**) Pictures of the three wax-patterned papers developed for RT-RPA on paper, obtained from this experimental set-up.*

Experiments in Guinea required a more portable instrumentation: all the components were anchored in a suitcase (Supp. Fig. 6a) and heavy technologies (UV lamp and sensitive camera) were replaced by lighter ones (LEDs and linear camera). The signal recorded by the linear camera, presented in Supp. Fig. 6b, consists in fluorescence intensity over a line of pixels. This carry-on equipment still requires a 220V voltage supply and a laptop for data recording, and costs around 10k€. However, several solutions have been already described in the literature to replace the fluorescent measurement by visual detection (Araujo, A.C. et al. Activated paper surfaces for the rapid hybridization of DNA

through capillary transport. *Anal. Chem.* **84**, 3311-3317 (2012) - Singleton, J. et al. Electricity-free amplification and detection for molecular point-of-care diagnosis of HIV-1. *PLoS ONE*, **9** (2014) - Song, Y. et al. Visual detection of DNA on paper chips. *Anal. Chem.* **86**, 1575-1582 (2014)).

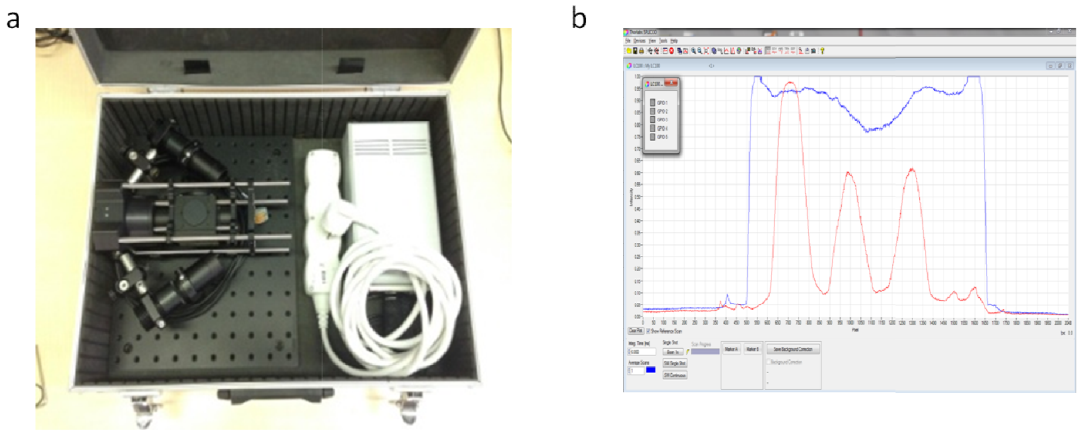

Supplementary Figure 6 *- Carry-on equipment used in Guinea to detect amplification on paper. (**a**) Picture of the experimental suitcase. (**b**) Raw data that can be extracted from the linear camera: fluorescent signal intensity over a line of pixels.*

In addition to technical specifications, one of the main difference between these two experimental sets-up lies in the signal localization. Because the laboratory equipment takes a full picture of the paper device, the measurement can be done over the entire microzone area or on a more local spot. With the carry-on device, the recorded signal highly depends on the vertical position of the detection line.

Experiments of RT-RPA amplification in long rectangular microzone are displayed in Supp. Fig. 7. The large size of the microzones produced an inhomogeneous spatial repartition of the signal. The laboratory set-up enables both global and local measurements. On the opposite, there is an important information loss in the case of the carry-on equipment.

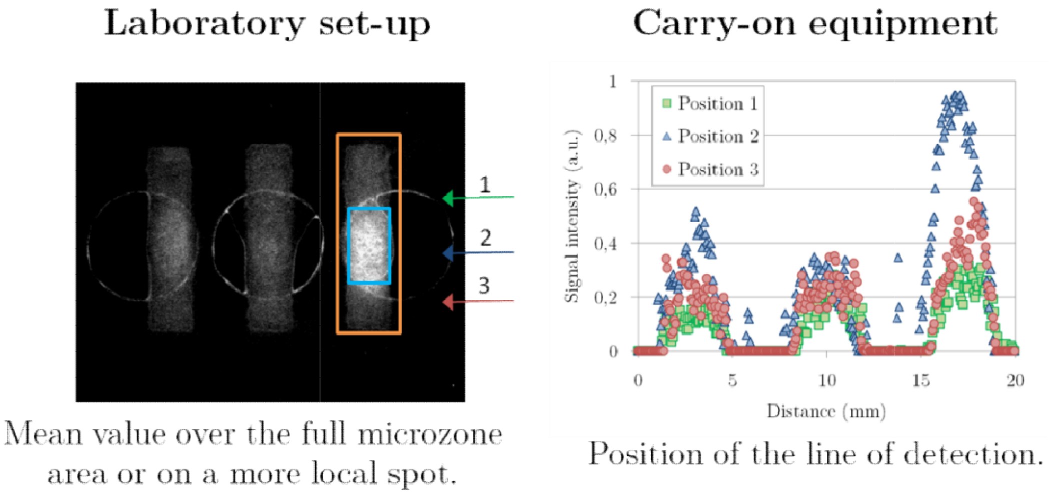

Supplementary Figure 7 *- Measure of an inhomogeneous signal on paper with each system of detection.*

With smaller microzones, the signal is usually more uniformly distributed over the paper. In these conditions, compared measurements from the two detection devices show good agreements, as displayed in Supp. Fig. 8. Indeed, after normalization, the fluorescent intensities measured with each experimental set-up, over each microzone from seven different papers, gathered towards the curve y=x.

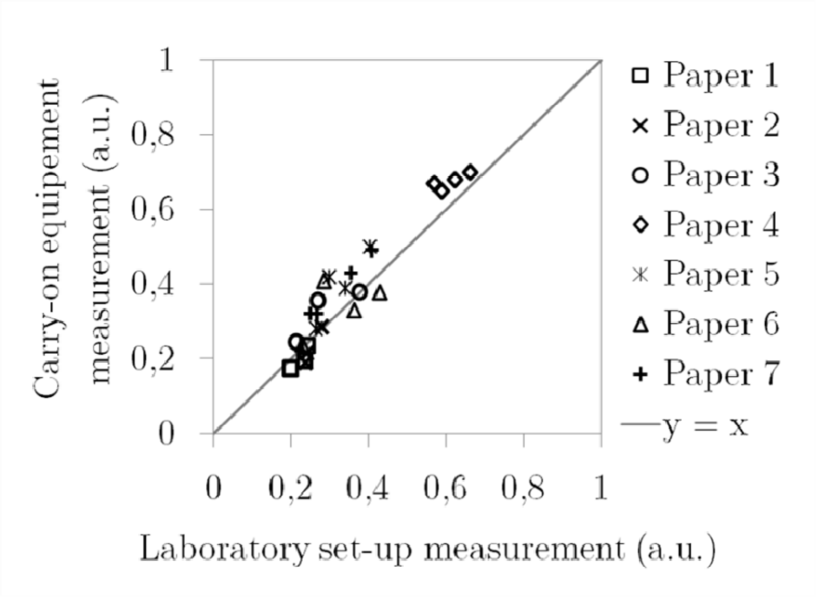

***Supplementary Figure 8** - Compared measurements of seven papers patterned with three to four microzones, from the two experimental sets-up.*

RT-RPA assay has the great interest to work at low temperature: between 37 and 42°C. Thus, a light and mobile heating device can be easily designed. We used a 9V-battery to power a conductive nickel pattern drawn on paper, around a fluidic device (Supp. Fig. 9a). According to the ambient temperature and the electric resistance of the conductive trajectory, a wide range of temperature can be reached: from 25 to 45°C (Supp. Fig. 9b). Transitory regimes only last a few minutes (data not shown).

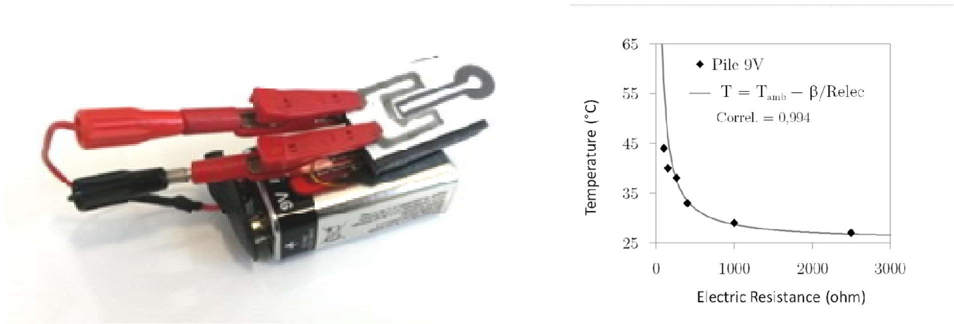

***Supplementary Figure 9** – Autonomous heating-device. **(a - left)** Paper device powered by a 9V-pattery through a conductive nickel pattern. **(b - right)** Temperature measured on the paper according to the electric resistance of the conductive trajectory.*

RT-RPA amplification on Guinean samples. Each RT-RPA amplification on paper, on viral extractions of Guinean samples, can be related to two internal controls that use freeze-dried synthetic small RNA strands. Thus, each experiment provides three amplification curves standing for the sample test, the positive control and the negative control.

We first analyzed controls by displaying the end point fluorescence intensity (after 20 minutes of experiment) in Supp. Fig. 10a. As a reminder, negative controls simply consist in RT-RPA reagents rehydration with water whereas positive

controls contain a fixed amount of synthetic RNA template (5µL – concentration: 10^7 copies/µL). A threshold is selected to maximize the number of well-identified controls: positive above and negative below the criteria. The distribution of the fluorescence intensity shows that negative controls constantly stay at a background level and 94% remain below the threshold. For positive controls, the parameter is more scattered and amplification seems to occur only in 66% of the experiments. In these conditions, the main cause of failure should be searched in the RNA stability or reagent degradation. With a total sample size of 136, the rate of combined successes between positive and negative controls on the same paper, reached 63%.

The sample tests analysis has been performed on the selection of the 43 paper devices for which positive and negative controls were well identified. All the biological samples contain RNA extractions. The challenge lies in the identification of the EBOV sequence. The nature of the content modifies the distribution of the measured parameter (difference of final fluorescence intensities between sample test and negative control) when compared with controls analysis, as shown in Supp. Fig. 10b. With the same previously defined threshold, 90% of the EBOV positive samples display a characteristic signal of amplification. However, the number of negative samples that remain at a background level is more modest (60.8%). RNA extractions seem to favor non specific amplifications in some cases.

These results reinforced the conclusions given by the ROC curves: contrarily to the case with synthetic RNA, our experiments on viral RNA display a good sensitivity but an insufficient specificity.

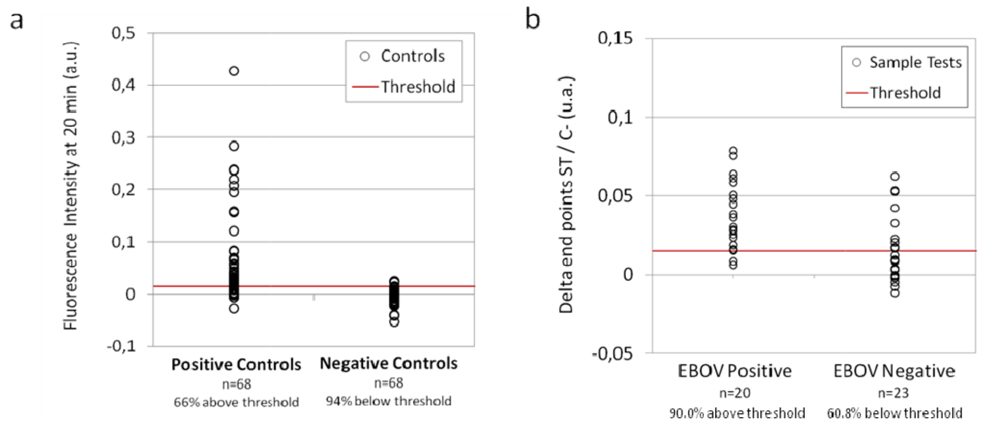

Supplementary Figure 10 *– Distribution of RT-RPA results for all experiments. (**a**) Final fluorescence intensity for all 68 positive and negative controls (black rings) and proportion of well identified results according to the threshold level (red line). (**b**) Difference of final fluorescence intensity between sample test and negative control for the 43 clinical sample experiments performed on paper with validated controls (black rings) and proportion of correct diagnostics for EBOV positive and EBOV negative according to the threshold level (red line).*
